# A patient and public involvement workshop using visual art and priority setting to provide patients with a voice to describe quality and safety concerns: Vitamin B12 deficiency and pernicious anaemia

**DOI:** 10.1111/hex.13152

**Published:** 2020-11-12

**Authors:** Natasha Tyler, Sally Giles, Gavin Daker‐White, Beth Clare McManus, Maria Panagioti

**Affiliations:** ^1^ NIHR Greater Manchester Patient Safety Translational Research Centre University of Manchester Manchester UK

**Keywords:** B12 deficiency, patient and public involvement, patient experience, patient safety, pernicious anaemia, PPI, primary care, priority setting, visual art

## Abstract

**Background:**

Patient and public involvement and engagement (PPIE) is recognized as important for improved quality in health service provision and research. Vitamin B12 deficiency is one area where PPIE has potential to benefit patients, as patients often report sub‐optimal care due to diagnostic delay, insufficient treatment and poor relationships with health professionals.

**Objective:**

In an effort to engage an understudied patient population in health‐care quality and safety discussions, and provide patients with an opportunity to have a voice, contribute to research priorities and express their current quality and safety concerns, we hosted a PPIE workshop.

**Methods:**

One researcher (with lived experience) facilitated a one day workshop with 12 patients with varied demographics. The workshop had four components (a) one‐to‐one sessions with an artist, (b) quality and safety research/education priority setting, (c) comments on research proposals, and (d) development of a PPIE group for future research.

**Results:**

All elements of the workshop elicited a number of quality and safety priorities for the group. Priority setting highlighted issues with interpretation of test results, symptom‐based treatment, self‐medication and relationship with primary care health‐care professionals. One of the major safety issues highlighted in the visual art element was feeling ignored, silenced or not listened too by health‐care professionals.

**Discussion:**

Visual art methods to express experiences of health, and research priority setting tasks achieved the aim of providing patients with an opportunity to have a voice and express concerns about health‐care quality and safety issues. The addition of visual art allowed patients to articulate emotions and impacts on everyday life associated with quality and safety.

**Patient or public contribution:**

A public contributor was involved in preparation of this manuscript. The event aimed to enable PPIE contribution in future research.

## 1 BACKGROUND

Vitamin B12 deficiency occurs when the body does not absorb adequate vitamin B12 from food or when there is not enough dietary intake of the vitamin.^1^ It is highly prevalent and despite the lack of consensus, UK estimates suggest it might affect around 6% of the population under 60% and 20% over 60.^1^, ^2^ Pernicious Anaemia is the most common cause of B12 deficiency in the UK^3^ but is not the only reason for patients becoming deficient in Vitamin B12. However, diagnosis of Pernicious Anaemia can be lengthy process, 14% of British patients with B12 deficiency in a recent survey waited over 10 years for a diagnosis of Pernicious Anaemia (PA).^4^

B12 deficiency can considerably affect patients’ quality of life and functioning. Clinical consequences include visual disturbances, memory loss, psychiatric abnormalities and loss of nerve function.^5^ Untreated vitamin B12 deficiency can result in anaemia, gastrointestinal disturbance or permanent neurological damage.^6^ Other common symptoms include tiredness and low mood.^2^

The most common approach to diagnosing B12 deficiency is a blood test although different blood tests are used (eg serum total cobalamin and serum holotranscobalamin) and there are also several other diagnostic approaches.^5^ The British Society for Haematology guidelines recommend that deficiency levels needed for a diagnosis are either based on manufacturers' reference ranges or determined by the individual laboratory using the test.^1^ Usual practice in the UK is treat B12 deficiency with injections. The injection is given intramuscularly with one injection every three months of vitamin B12, in a form called hydroxocobalamin.^7^

However, to qualify for treatment individuals must demonstrate deficiency in the diagnostic blood test, whereby ‘cut‐off’ rates and type of blood test used vary considerably between geographical areas or patients may show neurological signs of the deficiency without haematological signs.^1^, ^8^ A patient survey found that daily, weekly and monthly injections were associated with greater symptoms improvement than 2‐3 monthly injections.^9^ The current UK National Institute for Health and Care Excellence (NICE) guidance is that injections should be received every 8‐12 weeks (2‐3 months).^10^ A recent peer‐reviewed paper describes the impact of current guidance on patients

unfortunately, the vitamin B12 deficiency support groups are rife with individuals forced to self‐inject to keep their symptoms at bay, while their physicians tell them their B12 levels are normal.^9^

Current practices for diagnosing and treating B12 deficiency raise major but overlooked patient safety concerns which contribute to health inequalities and marginalization of individuals with B12 deficiency. A recent UK survey found that two‐thirds of individuals with PA who received treatment for their deficiency were dissatisfied.^4^ Qualitative work often uses war and battle metaphors to describe the struggles these patients face in primary care settings such as ‘The struggle to achieve a diagnosis’, ‘The significance of a diagnosis’ and ‘Battling for sufficient treatment’.^11^ Patients have also described feelings of stigma, dissatisfaction with their medical care due to diagnostic delay, insufficient treatment and poor relationships with health professionals.^11^ Many experienced, anticipated, and internalized stigma, which led to a reduced quality of life and withdrawal from medical care. Stigma can be categorized as felt (the shame and expectation of discrimination that prevents people from talking about their experiences) or enacted (the experience of unfair treatment by others).^12^ Patients with this condition report both, primarily due to the vague symptoms (i.e. fatigue, concentration issues).^11^

### 1.1 Aim

Existing evidence highlights a dissatisfaction with current treatment and diagnosis practice and policy in primary care; which is likely to raise quality and safety concerns. This paper reports a patient and public involvement workshop that aimed firstly to understand and consolidate quality and safety for this patient group concerns using numerous verbal and written methods and secondly to assess any additional impact of using visual art as a means of expressing quality and safety concerns.

We use NIHR definitions of Public and Patient Involvement and Engagement (PPIE),^13^ whereby public involvement in research involves ‘working with researchers to develop or comment on research materials, providing advice as members of a project steering group’. From an involvement perspective, this work aimed to co‐develop priorities for future quality and safety research projects and to develop a PPIE group/steering committee for future research. From an engagement perspective, this work aimed to raise awareness of quality and safety research and enable participants to consider their experiences in terms of quality and safety.

## 2 METHODS

### 2.1 Attendees

Participants were contacted and invited to attend the workshop via a regional mailing list from a pernicious anaemia charitable organization, university volunteer newsletters and social media (Facebook support group). Participants were asked to self‐identify and email the researcher if they were interested. Due to limited funding, we had space for 13 participants at the event, recruitment was on a first to identify basis. In order to be included participants must have a diagnosis or be receiving treatment for Vitamin B12 Deficiency or Pernicious Anaemia, there were no restrictions on the causes of B12 deficiency. Twelve participants attended the workshop from a demographically diverse group ranging from teenagers to older adults, multiple ethnicities and both genders were representd. One participant could not attend on the day due to illness. As this was an engagement event and not research demographic data was not collected, and whilst others attended the most highly represented group were caucasian females aged 25‐50; this may reflect that the condition may be more common amongst this group.^14^ No participants had previous experience of patient and public involvement in vitamin B12 deficiency research.

### 2.2 Settings and design

A workshop titled ‘B12 Deficiency/Pernicious Anaemia Patient Safety Workshop?’ was held in March 2020. The workshop had four components (a) one‐to‐one sessions with an artist, using visual art as a means to express experiences of health (b) research/education priority setting activities in relation to health‐care quality and safety (c) comments on research proposals (d) development of a patient and public involvement group for future research.

### 2.3 Procedure

Participants were informed at the beginning of the workshop (verbally and in writing) that this was a discussion to gather their views on quality and safety, involvement of patients in research and an opportunity to work with a patient experience artist. Participants were informed how the information and creative outputs they provided would be used.

**Figure 1:**
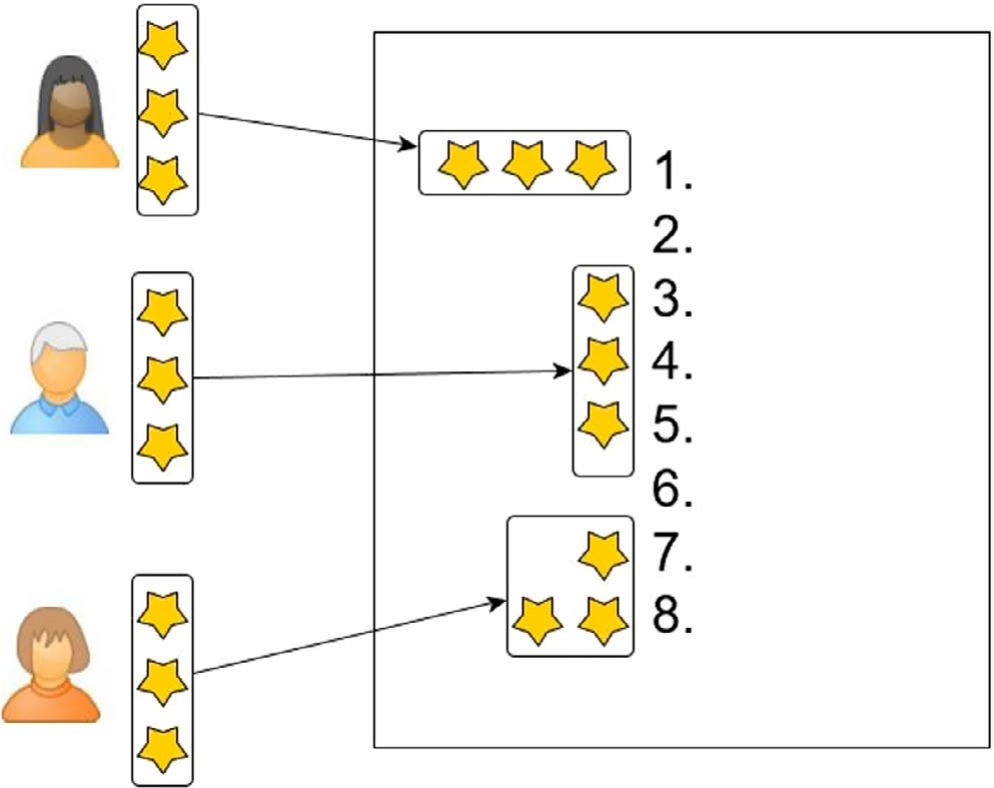
A figure to illustrate ways participants might use their stickers in the ranking task to highlight priorities

As the workshop aimed to understand quality and safety issues we used many conventional methods for gathering such information in PPIE workshops. This included various discussions: (a) discussions of proposed research, (b) patient safety in B12 deficiency, (c) priorities for health professional education, (d) future research and practice priorities. In terms of priorities for education and patient safety, the discussions were designed to generate the participant's ideas around these topics (rather than to present and discuss existing ideas), the aim was to provide a clean slate for participants to develop their own priorities. Following presentations and discussions on each topic, participants worked in groups of 2‐3. Group discussions were facilitated by the host (NT). Following small group discussions, there were opportunities to feedback to the whole group as the facilitator took notes. After two discussions, lists were drawn up that captured the group's key thematic concerns (patient safety priorities and education priorities) followed by two ranking tasks. Participants were each given three stickers and asked to choose the items most important to them (prompts such as ‘What would you like future research to address?’ ‘What would you like health professionals to be educated about/receive training in?’ were used to help participants decide. Participants were able to put all stickers on one item or spread them across three, see Figure 1.

In addition to more traditional methods of generating and consolidating quality and safety concerns, we used a novel method of visual art, whereby participants worked one‐on‐one with an artist. Visual art has been used to help respondents consider health matters, in numerous ways, for example in therapy,^15^ or illness meaning‐making,^16^ but rarely to highlight quality and safety concerns and priorities. During the workshop, participants were invited to one‐to‐one sessions with a patient experience artist, who took portrait photographs of the participants (also hands and back of head shots of participants that wanted to remain anonymous). After the photographs were printed, participants then worked on acetone to develop artwork that expressed their concerns about their condition and health‐care quality and safety. Participants were also asked to bring an item that summarized their patient experience to be photographed. As this happened throughout the day, the creative activities were used as a separate output to the priority setting outputs (not woven into discussions). Both will be used to inform future research within our team and it is hoped the images/outputs can also have a secondary purpose and be used for educational purposes to highlight sub‐optimal patient experiences and patient perspectives to health‐care professionals.

## 3 RESULTS

### 3.1 Patient safety priorities

Table 1 shows the list of health‐care quality and safety priorities generated by the participants and the order of their importance (indicated by the number of votes) in the ranking task (see Table 1). The primary patient safety priority proposed by patients was the need to improve interpretation of test results and reduce inconsistencies in reference ranges. Participants described national and local inconsistencies whereby initiation (and sometimes frequency) of treatment is dependent on reference ranges of test results set by local test centres instead of a national standard test or reference range (Table 1). In relation to this, the second most important priority for patients was that general practitioners (GPs)/health‐care professionals should overcome this by adopting a symptom‐based approach. Third, participants stated that they were aware of individuals that chose to self‐medicate and felt this was a major safety concern for several reasons and they often did not inform their GPs. Thus, patients might use medication which is not always obtained from secure sources or following validated advice and as a consequence infections/safety complications associated with injection could occur. A lack of trust in health‐care professionals was an equally important safety concern for this group.

**Table 1:** Patient safety priorities/concerns for B12 deficiency and pernicious anaemia patients

No	Patient safety issue/priority	Votes
1	Interpretation of test results and inconsistent reference ranges	8
2	The necessity for symptom‐based treatment (less reliance on tests)	6
3	Safety of self‐medication	5
4	Lack of trust in General Practitioners (GPs) and health‐care professionals	5
5	General practitioners variable knowledge about treatment and tests	
	Feelings of disempowerment for patients	3
7	Diagnosis: GP reluctance to test further, but safety and treatment benefits associated with pernicious anaemia diagnosis	2
8	Time to diagnosis (too long)	2
9	Postcode lottery	2
10	Safety of social media as a source of information, advice and support	1
11	Insufficient information about pre‐blood test behaviour (fasting)	0
12	Ineffective testing following treatment commencement	0

### 3.2 Educational messages to health‐care professionals

In the second ranking task, participants were asked to describe the key messages they would like to portray to health‐care professionals about their experiences. Participants were not asked to consider this specifically from a quality and safety perspective. Thirteen messages were produced and four messages received the same amount of votes (see Table 2). The four top‐ranked messages were (a) treatment is too infrequent‐ participants felt that treatment needs to be more frequent and based on symptoms rather that tests; (b) GPs should better recognize and understand symptoms—participants felt that GPs did not understand their symptoms; (c) lack of awareness of day‐to‐day effects of the condition—participants felt that health‐care professionals did not understand the scale of the effect of the condition on their quality of life and (d) include test for B12 in standard blood test—participants felt that B12 tests should be included in standard blood tests, to identify patients with the condition timely.

**Table 2:** Patient messages to health‐care professionals

No	Patient messages to health‐care professionals	Votes
1	Listen to patients when they say treatment is too infrequent	4
2	Necessity for General Practitioners to recognize and understand symptoms	4
3	Lack of awareness of the emotional and day‐to‐day effects of the condition	4
4	Include tests for B12 levels in standard blood test	4
5	Listen to patient (don't discount because symptoms are wide ranging, vague and invisible)	3
6	Understand co‐morbidity of B12 deficiency and other conditions (IBS, depression, vitamin deficiencies)	3
7	Please take condition seriously	2
8	The effect of social media on patient experience and behaviour	1
9	Gain greater knowledge of co‐factors associated with B12 deficiency	1
10	Don't discount the patient experience or their own research	1
11	Recognize that health‐care professionals can't know everything about every condition	0
12	Educate all health‐care professionals about B12 deficiency and blood tests	0
13	Awareness that the UK blood test range is very low (compared to other countries)	0

### 3.3 Creative outputs

**Figure 2:**
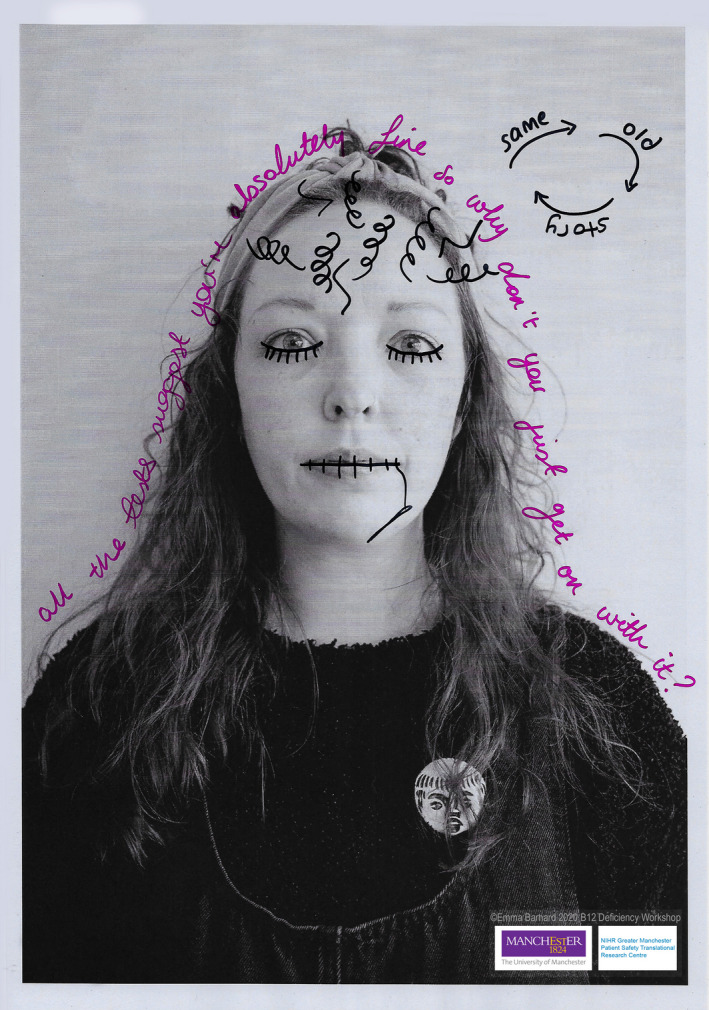
An example of a portrait to highlight a participants negative experiences of primary care interactions

**Figure 3:**
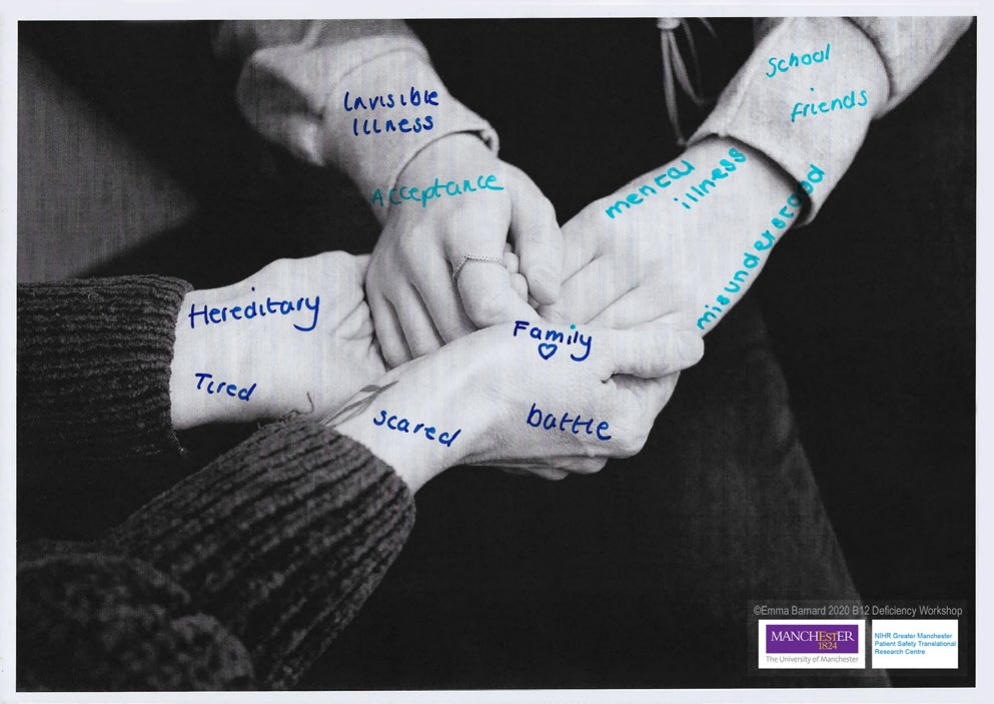
An example of visual art created by a mother and daughter who both suffer vitamin B12 deficiency

All participants generated at least one visual/creative output, many generated numerous outputs, Figures 2 and 3 show examples of this (full online gallery can be accessed here: https://b12patientsafety.weebly.com/). Many of the portraits highlighted themes of disempowerment in health‐care consultations, not feeling listened to or stigmatization. Others highlighted symptoms of the condition visually, such as brain fog. In terms of the items that participants chose to bring, one participant baked a cake portraying a graveyard (highlighting the ‘death sentence’ she felt to be associated with the condition). Many participants brought sharps boxes to highlight their inclination to self‐medicate due to not feeling listened to by health professionals or supported by policy and practice. Others brought items that helped alleviate their symptoms (ie eye spray).

Feedback from the work with the artist was generally positive. When asked about the benefits of using visual art as a means of eliciting quality and safety concerns, participants felt that visual outputs had more potential to have impact in terms of changing practice. Participants often described how they felt that health‐care professionals would not listen to their individual concerns or take them seriously, but felt the collective effect of lots of visual displays of many patients with the same condition, may be more likely to highlight the poignancy of these issues to patients. Others felt it provided a novel opportunity to reflect on their personal feelings towards their condition. Some valued the opportunity to explore and discuss their perspectives with other patients; which the artistic element helped facilitate. A selection of quotes from participants are presented below:

People connect with people. Using the strong visuals and personal stories is the key to getting an audience with a listening ear

Working with an artist to give a visual image to show our illness and treatment is more powerful than our invisible ignored words

It has been beneficial to meet the artist and to stand away from myself and to try and present a visual image of how I feel about my illness

It has been such a positive experience for myself and my daughter today with the artist. A totally unique and different way of portraying how we feel. It is inspiring to know we are not alone.

The workshop enabled patients to express themselves with regards to their condition. I am leaving here today feeling that door has been opened to find a way for health professionals to hear patient voices. From a patient perspective, it was interesting for me to find out how similar our experiences are. This demonstrates that the issues raised are likely to be systematic.

I do believe this work could have impact and feed into NICE and BNF guidelines

## 4 DISCUSSION

Visual art provided a means of articulating quality and safety concerns that could be missed in more traditional PPIE methods (discussions, ranking exercises). However, the main benefits for participants seemed to lie in impact, participants felt the visual outputs have greater potential impact than words in terms of highlighting their experiences and changing the practice of health‐care professionals. Participants also described the interaction of shared experiences with a group and visual art, as a means of reflecting about and articulating their quality and safety concerns and experience as a patient.

This workshop showed that patients with B12 deficiency expressed serious concerns related to health‐care quality, safety and treatment. The priority setting element of the workshop suggested that most serious patient safety concerns were the interpretation and reliance on sub‐optimal biological tests (as opposed to symptom‐based treatment), concerns around the prevalence of self‐medication and sub‐optimal relationships with primary care professionals. Most participants were keen to address issues around the safety of treatment practices and self‐medication as key priority. Other suggestions focused on the effects of their condition and current health‐care policy and practice on quality of life, relationship with health‐care professionals and stigmatization (health‐care professionals, friends and family not understanding the effect of symptoms on quality of life and functioning). However, the visual art exercise instead highlighted feeling disempowered with one's health and access to care and not being listened to as the most predominant themes. Much of the feedback in the visual art related to self‐validation and illness self‐identity; which is in line with safety concerns in wider primary care patient safety literature.^17^

As B12 deficiency and pernicious anaemia affect a relatively large proportion of the UK population,^1^ these concerns have to be documented and addressed using high‐quality research. In an understudied population, the involvement of patients in all stages of research is important to identify and prioritize relevant areas of research. Participants were keen to contribute to future research and expressed a willingness to be included in all stages of the research process. They also highlighted the importance of expressing the patient voice primarily to health‐care professionals as means of influencing policy and practice. Participants also thought that the creative outputs are a powerful means of communicating their illness experience with health providers and members of the public. They hoped that the use of creative outputs has the potential to highlight inequalities that they encounter whilst trying to access medical treatment for their symptoms and the lack of emotional compassion that they often experience by health‐care providers, family members/friends and the wider public community.

This workshop has confirmed some of the findings of previous research, by also highlighting the feelings of stigmatization and ‘a battle for adequate treatment’.^11^ This workshop, however, has approached this issue via a health‐care quality and safety perspective. As patient safety has expanded in recent years from being confined to hospital medicine to widening to include primary care settings and other settings.^17^, ^18^ Many of the health‐care safety concerns expressed by this population are similar to those of other clinical groups. Patient safety literature in primary care is predominantly related to the characteristics or behaviour of patients, staff or clinical systems and interactions between staff and patients and or people and systems. In primary care, patient safety is often expressed as a subjective feeling or judgement grounded in moral views and with potentially hidden psychological consequences affecting care processes and relationships.^17^ This is particularly poignant for patients with B12 deficiency and pernicious anaemia, who consider safety in terms of sub‐optimal policies and systems and relationships with health‐care providers. Whilst these are not necessarily patient safety incidents or events reported, in this workshop, the themes described are in line with contributory factors to patient safety in primary care (communication, training, dignity, respect).^17^, ^18^

Photographs and creative outputs to give patients a voice have been used successfully in previous research.^19^, ^20^ Literature suggests that using photographs as a means of expression enables patients with mental health conditions to highlight issues that are difficult to put into words.^19^ This workshop shows that creative patient expression is also effective for patients with physical illnesses. Photography and creative outputs provide a voice to patients with invisible illnesses, many of whom who felt invisible to health‐care professionals. Participants described how they felt empowered to participate in future research, were optimistic that the methods used have potential to generate change in policy and practice, and enjoyed the opportunity to express themselves and share their experiences with others.

### 4.1 Learning points

The main learning point of this event was that for clinical conditions that have a great impact on quality of life, expressing quality and safety concerns is amenable to visual art representation. Many of the outputs depict patient experience in a more poignant way than words alone and the predominant issues highlighted differed somewhat from issues highlighted through traditional methods (discussions, ranking tasks). The main difference between perceptions of the methods from participant perspective was the perceived impact of the visual art outputs to be used beyond the event for educational purposes to influence policy and practice change.

Whilst commissioning an artist to work with patient groups at all future PPI workshops might not be financially viable, this workshop shows how important it is to have a variety of accessible options for expressing oneself, patients very much enjoyed the opportunity to be completely creative with their expression, for example baking and decorating a cake for the event. Options to be creative, such as giving participants the option to bring an object to the workshop, photography or drawing might be useful with clinical populations or individual that inherently suffer communication problems or lack confidence in group discussions. Such additions to PPI workshops do not necessarily need to be costly. However, creative options should not be enforced, but an option for those interested.

Often researchers use PPIE workshops exclusively to gather comments on existing project ideas; however, this workshop highlighted that the most valuable process was enabling participants to consider their own quality and safety concerns using a ‘blank slate’ rather than presenting predetermined research ideas. Participants were not‐critical of any elements of the proposed research plans, so this did not generate any novel perspectives or outputs, this speaks directly to a Lancet series ‘increasing value reducing waste’^21^ whereby in an understudied population, patient involvement in research is pertinent at the outset, to ensure that the research community addresses the questions that matter most to the population. As a result, we have adapted our future research proposals based on the priority setting at this event, rather than use the direct feedback on research proposals; which was non‐critical (rather than use the proposed measure in the questionnaire research we planned, we have a found another measure that is more in line with patients priorities expressed in the visual art outputs).

### 4.2 Strengths and limitations

One strength of this workshop was that it offered a different means of participation for participants that do not enjoy speaking openly in a group. One‐to‐one engagement with an artist gave quieter members of the group and opportunity to reflect and also describe their patient experience. Visual art methods also provided tangible outputs that can be used for educational purposes. Participants considered these impactful; ensuring participants consider the workshop outputs impactful is important when participants are giving‐up their free time to attend and it may have positive implications regarding likelihood of future PPIE engagement. The study was limited, as we did not collect demographic information (including diagnosis) about the participants, there may have been distinct differences between those with Pernicious Anaemia diagnosis and those with vitamin B12 deficiency for other clinical reasons. Also, there may have been bias in terms of representation of patient experiences due to recruiting from social media. We did not use robust qualitative synthesis methods or conduct robust analysis of the data/information recorded at this workshop, as it was deemed PPIE. Whilst nobody with accessibility issues volunteered to attend the event, it may not have been accessible to everybody with this condition and future workshop hosts might want to consider remote engagement options too.

## 5 CONCLUSION

The workshop highlighted that patients with B12 deficiency experience important health‐care safety and quality issues; which negatively affect satisfaction with treatment, quality of life, feelings of stigmatization and management of illness. In more traditional verbal and written methods, patients indicated that their key health‐care safety priorities are related to treatment policy and practice, and self‐medication. However, the visual outputs focused on self‐validation and illness self‐identity; which are often contributory factors to patient safety. This workshop highlighted that patients with invisible illnesses are keen to have their voice heard and engage with novel approaches, particularly if they generate outputs that have potential to impactful alongside any research findings. Working with an artist presented a novel opportunity for patients with B12 deficiency to articulate quality and safety concerns, and also generate outputs that might educate health‐care professionals about stigmatization and sub‐optimal patient experience As a result participants were keen to get involved in future research and generally enjoyed the workshop.

## CONFLICT OF INTERESTS

The authors declare that they have no competing interests.

## AUTHOR CONTRIBUTION

The original idea for the workshop was developed by NT and MP. SG provided expertise about PPI workshops and how to structure the event. NT wrote the paper and all authors (GDK, MP, SG, BCM) contributed to critical revision of the manuscript. BCM was a public contributor who attended the workshop and helped write the manuscript. All authors read and approved the final manuscript.

## ETHICAL APPROVAL AND CONSENT TO PARTICIPATE

Reviewed by Social Responsibility Senior Management Team at the University of Manchester and as the work constituted PPI it was not deemed to require UREC review.

## Data Availability

Data is available upon reasonable request.
